# Mapping Pitch Accents to Memory Representations in Spoken Discourse Among Chinese Learners of English: Effects of L2 Proficiency and Working Memory

**DOI:** 10.3389/fpsyg.2022.870152

**Published:** 2022-05-19

**Authors:** Connie Qun Guan, Wanjin Meng, Laura M. Morett, Scott H. Fraundorf

**Affiliations:** ^1^School of Foreign Studies, Beijing Language and Culture University, Beijing, China; ^2^Department of Psychology, Carnegie Mellon University, Pittsburgh, PA, United States; ^3^China National Institute of Education Sciences, Beijing, China; ^4^Department of Educational Studies in Psychology, Research Methodology, and Counseling, University of Alabama, Tuscaloosa, AL, United States; ^5^Department of Psychology and Learning Research and Development Center, University of Pittsburgh, Pittsburgh, PA, United States

**Keywords:** L2 processing, pitch accent, discourse, memory, working memory

## Abstract

We examined L2 learners’ interpretation of pitch accent cues in discourse memory and how these effects vary with proficiency and working memory (WM). One hundred sixty-eight L1-Chinese participants learning L2-English listened to recorded discourses containing pairs of contrastive alternatives and then took a later recognition memory test. Their language proficiency and WM were measured through standard tests and the participants were categorized into low, medium, advanced, and high advanced language proficiency groups. We analyzed recognition memory task performance using signal detection theory to tease apart response bias (an overall tendency to affirm memory probes) from sensitivity (the ability to discern whether a specific probe statement is true). The results showed a benefit of contrastive L + H* pitch accents in rejecting probes referring to items unmentioned in a discourse, but not contrastive alternatives themselves. More proficient participants also showed more accurate memory for the discourses overall, as well as a reduced overall bias to affirm the presented statements as *true*. Meanwhile, that the benefit of L + H* accents in rejecting either contrast probes or unmentioned probes was modulated for people with greater working memory. Participants with higher WM were quite sure that it did not exist in the memory trace as this part of discourse wasn’t mentioned. The results support a contrast-uncertainty hypothesis, in which comprehenders recall the contrast set but fail to distinguish which is the correct item. Further, these effects were influenced by proficiency and by working memory, suggesting they reflect incomplete mapping between pitch accent and discourse representation.

## Introduction

A general challenge for the second language (L2) learners is learning to associate linguistic forms with meaning at different levels of linguistic representation (Perfetti, 1997). Thus, a central scientific issue in the study of L2 learning is whether and to what extent learners can exploit these associations in their L2 well enough to achieve native-like performance in learning and memory. More generally, there is a debate as to whether L2 processing is qualitatively different from L1 processing [e.g., Marinis et al. (2005); Papadopoulou (2005), Clahsen and Felser (2006), and Felser and Roberts (2007)] or if native-like performance can be obtained with increased L2 proficiency or exposure [e.g., French-Mestre (2002), Hopp (2006, 2010), Jackson (2008), and Jackson and Dussias (2009)].

The goal of the current study aims to examine the individual differences effects on the prosodic memory trace when the discourse contains high vs. low PA contrast vs. non-mentioned alternatives. Herein, we examine these questions in the domain of speech prosody, an important cue to sentence and discourse processing (Cutler et al., 1997; Wagner and Watson, 2010). L2 listeners adopt different prosodic processing strategies or mechanisms than L1 listeners (Pennington and Ellis, 2000; Akker and Cutler, 2003; Baker, 2010; Braun and Tagliapietra, 2011). When prosodic cues match the content of speech, they enhance L2 speakers’ discourse comprehension and memory (Lee and Fraundorf, 2019, 2021). For L1 speakers, when prosodic cues fail to match the content of speech, they interfere with discourse content and memory (Harrington, 1992; van den Noort et al., 2006; Morett and Fraundorf, 2019; Morett et al., 2020, 2021). At present, it is unclear whether this is the case for L2 speakers, however.

In the present study, we examine how differences in prosodic cues affect how L1 Chinese learners of L2 English map prosodic pitch accents (PAs) to discourse status and how it affects comprehension and memory for spoken discourse. We also consider the contributions of language proficiency and working memory (WM) to the impact of PA on discourse comprehension and memory.

We focus on L2 learners’ acquisition of the mapping of different PAs to representation of spoken discourse as we all know that, learning to comprehend PAs in spoken discourse is a practical issue for L1-Chinese learners of L2-English. Nevertheless, the mapping between PAs and semantic integration in discourse has long been neglected in L2 teaching and learning practices (Gut and Milde, 2002; Gut, 2003; Gut et al., 2007; Braun and Tagliapietra, 2011). Therefore, learners with limited L2 proficiency are likely to fail to make use of PA in L2 listening comprehension, leading to poor comprehension and memory. This void in both research and practice requires further scientific investigation on whether and to what extent L2 learners can map PAs to discourse representations in learning and memory.

### How Do Pitch Accents Affect Memory in L2 Processing?

Previous work (Lee and Fraundorf, 2017) found that L1 Korean learners of L2 English showed substantial differences from L1 English monolinguals in how they used PA information to encode and remember a discourse. In the present study, we aimed to investigate whether similar L1-L2 differences exist among L1 Chinese learners of L2 English. Below, we review three hypotheses about how contrastive PAs might affect language comprehension and memory: granularity, contrast representation, and contrastive uncertainty. First, the *granularity* account claims that the effect of a contrastive PA, and perhaps focusing devices more generally, is to enhance the representation of the accented word itself. This account was originally proposed to describe the mnemonic benefit of other focus-marking devices, including *it*-cleft constructions (Just and Carpenter, 1987, 1992; Birch and Garnsey, 1995; Sturt et al., 2004) and font emphasis in a written discourse (Sanford et al., 2006), but could also describe effects of PAs (Norberg and Fraundorf, 2021). The granularity account predicts a contrastive PA should enhance a listener’s ability to reject all false alternatives because all of those are inconsistent with the correct information.

Alternatively, the *contrast representation* account proposes that the mnemonic benefit of contrastive PAs over presentational PA is that contrastive PAs enhance the representation of specific salient alternatives in the discourse. For example, in discourse (1, 2) above, the *British* scientists are contrasted with the *French* scientists, so a contrastive PA may lead listeners to retain something about the contrastive *French* scientists in particular. This account is supported among L1 listeners by Fraundorf et al. (2010), who found that a contrastive PA facilitated comprehenders’ later ability to correctly reject the salient alternative, but not of wholly unmentioned items never part of the contrast set.

Thirdly, the *contrastive uncertainty*^1^ account refers to the possibility that comprehenders encountering a contrastive PA may bring to mind the set of contrasting alternatives but fail to encode which is the correct statement and which is the alternative. After all, some degree of uncertainty about the linguistic input is a fundamental characteristic of language processing [e.g., Goodman and Lassiter (2015)]; comprehenders may remember that there was a contrast between two alternatives (such as *British* and *French*) but be uncertain about which member of the set was referred to later. In this case, a contrastive PA may thus confer no benefit on, or even counterintuitively *impair*, the ability to rule out the salient alternative. By comparison, a strong memory representation of the two mentioned alternatives might benefit comprehenders’ ability to reject the unmentioned-item probe. That is, the correct and the contrastive alternatives are easy to tease apart among L1 listeners, but not among L2 speakers because both pieces of information exist in L2 listeners’ memory with less degree of certainty about which was originally stated. This is what has been found with L1-Korean speakers learning L2-English (Lee and Fraundorf, 2017, 2021), suggesting that L2 learners do not process PA cues the same way as native speakers.

However, given the influence of L1 on L2 processing (Rasier and Hiligsmann, 2009; Mennen, 2015), it is unclear whether this pattern is unique to L1-Korean speakers or whether it characterizes L2 prosodic processing more broadly. Here, we sought to determine whether a similar pattern of L2 prosodic processing emerged among L1 Chinese speakers learning L2 English.

### Pitch Accents as Cues to Discourse Processing

PA represents a point of both commonality and difference between Chinese and English. On the one hand, both Chinese and English have tones anchored on stressed syllables, i.e., lexical tones in Chinese and PAs in English (Duanmu, 2004). On the other, the function of these tones differs drastically. English has no lexical tones, and so its words can take different PAs to express contextual meanings, such as marking a focused entity. By contrast, Chinese tones are lexically contrastive. Instead, focus on Chinese is conveyed by providing lexical cues or by putting stress at the end of the syllable *via* pausing, lengthening, rising, or falling tones (Ouyang and Kaiser, 2012; Yang and Chen, 2014, 2018; Lee et al., 2015, 2016; Wang et al., 2020). Hence, understanding the focus information conveyed by PAs in English could be a great challenge for Chinese learners of L2 English.

Pitch accents are phonological constructs that are placed on particular words and are usually realized acoustically with longer duration, greater intensity, and greater pitch excursion than unaccented words [for review, Ladd (2008)]. Most theories of prosody distinguish multiple types of PAs. Here, we focus on the distinction between contrastive PAs and presentational PAs, denoted as L + H* and H*, respectively, in the ToBI system for intonational transcription of English (Silverman et al., 1992; Beckman and Elam, 1997). Specifically, the L + H* and the H* accents both indicate a salient tonal target (an H*) on stressed syllables, but they are distinct from each other in that, in the L + H* accent, the salient tonal target involves a steep rise from an initial low tone (L), similar to Mandarin Tone 2, whereas in the H* accent, the salient tonal target remains flat, similar to Mandarin Tone 1. The L + H* accent has been considered to correspond to information that specifically contrasts with some other information in the discourse, whereas the H* accent accompanies new information more generally (Pierrehumbert and Hirschberg, 1990).

Thus, understanding PAs can be crucial for interpreting a sentence or discourse at a semantic and pragmatic level. Indeed, PAs contribute to native listeners’ initial online processing of spoken discourse. Experiments using the visual-world paradigm suggest that not only are PAs rapidly detected, but they can also be integrated into the discourse representation in the first moment of processing (Dahan et al., 2002; Ito and Speer, 2008; Watson et al., 2008).

Further, PAs contribute to long-term memory for a spoken discourse [e.g., Fraundorf et al. (2010, 2012), Gotzner et al. (2013), and Lee and Snedeker (2016)]. We highlight a particular study by Fraundorf et al. (2010) because it is most relevant to our present design. Fraundorf et al. (2010) examined memory for spoken discourses, such as (1, 2) below. A context passage (1) first established two contrasts, each between a pair of items (e.g., *British* vs. *French* and *Malaysia* vs. *Indonesia*). A continuation passage (2) then picked out one item from each contrasting set. The pitch accent on each of these critical words was manipulated between a presentational or contrastive accent through splicing.

(1) Both the British and the French biologists had been searching Malaysia and Indonesia for endangered monkeys.
(2) Finally, the (British/BRITISH) spotted one of the monkeys in (Malaysia/MALAYSIA) and planted a radio tag on it.

After listening to all the recorded stories, participants completed a recognition memory test for the referent chosen in each continuation. Participants were presented with probe statements, such as (3), and had to indicate whether each statement was true or false. The probe statements could refer either to the correct item (e.g., *British*, a true statement that should be affirmed), to the contrastive alternative from the original discourse (e.g., *French*, a false statement that should be rejected) or a wholly unmentioned item (e.g., *Portuguese*, a false statement that should be rejected).

(3) The (British/French/Portuguese) scientists spotted the endangered monkey and planted a radio tag on it.

Fraundorf et al. (2010) found that when the critical word was originally heard with a contrastive PA, memory was more accurate even a day later. Critically, this effect came about specifically because contrastive PAs facilitated rejection of the contrast item (e.g., *French* in example 3 above); contrastive PAs did not benefit rejections of an unmentioned item that was never part of the contrast set (such as *Portuguese*). Thus, Fraundorf et al. (2010) concluded that contrastive PAs lead comprehenders to encode and remember a salient alternative to the accented item, consistent with linguistic theories that posit the role of contrastive focus is to introduce a set of salient alternatives into the discourse [e.g., Rooth (1992)].

### Processing Pitch Accents in L2

However, recent research suggests L1–L2 differences frequently emerge in PA processing even under circumstances otherwise favorable for L2 processing. For instance, Akker and Cutler (2003) found that L1 Dutch listeners of L2 English could only detect focused information, and not contrastive information, despite the similarity of the two languages in their prosodic systems. Similarly, even highly proficient non-native listeners could not distinguish idiomatic from non-idiomatic expressions in English when marked with prosodic cues (Vanlancker-Sidtis, 2003).

With respect to the above memory task probing the effect of prosody on long-term discourse representations, Lee and Fraundorf (2017, 2021) found dramatically different patterns among L1-Korean college students learning L2-English. Low- and mid- proficiency learners (as defined by scores on a cloze task) showed no memory benefit whatsoever from contrastive PAs. High-proficiency learners were sensitive to contrastive PAs, but even they processed them in a non-native-like way: The contrastive PAs did not help them rule out a contrastive alternative and in fact, led participants to falsely affirm the contrastive alternative more often. Rather, contrastive PAs helped the high-proficiency L2 learners to reject items completely unmentioned in the discourse. This pattern suggests that the high-proficiency L2 learners did encode a set of contrasting alternatives in response to the contrastive pitch accent (which would help them reject the unmentioned alternative), but they failed to correctly encode *which* alternative was the correct one.

Considering the similarities in tonal features in Chinese and Korean L1, these differences are likely to be even starker for L1 Chinese speakers learning L2 English because of the dissimilarity of their prosodic systems. Chinese is a tonal language centered on each character and each character matches with the correct tonal information that represents a meaning unit. Different meaning units plus pitch accents will construct a sentence structure. Chinese tones are fixed to each Chinese character, which suggests that sentences with different tones can be spoken accurately as long as each Chinese character and its tone is pronounced, and tones are correct. The same syllable, when it is spoken with four different tones, can have four distinctive meanings (Wang et al., 2008). For example, /shu/1 uncle, /shu/2 ripe, /shu/3 summer, /shu/number. There are two basic types of Chinese tone trends in general: falling tone and rising tone. Chinese tones and intonation work together, but tones do not change with the intonation. Korean is an alphabetic language (Kim et al., 2016), which is similar to English. Each of its vowels and consonants has no tone. Standard Korean also does not have a system of using stress to distinguish the meaning of words. The most basic tone in Korean is like a curve that bulges out in the middle. That is, it starts with a low tone that goes up and then goes down. This is the same as in English. Korean tone is characterized by the number of syllables, which is about 2,000 (Taylor and Taylor, 2014). This is the same as Chinese, but there is a difference in the number of syllables, and Korean has a richer phonological system than Chinese. Chinese has about 400 syllables, which is much less than Korean (Taylor and Taylor, 2014); the first two syllables and the last two syllables have low-high intonation, and when the first one is difficult to pronounce, “low-high-low-high” becomes “high-high-low-high.” The intonation of the phrases in the sentence changes according to the intention of the speech, which is also similar to Chinese.

In general, the L1–L2 relationship seems to affect the acquisition of L2 prosody, which in some cases can make L2 prosody more difficult to process. For example, Rasier and Hiligsmann (2009) found a relationship between the typological distance between the learner’s L1 and L2 (markedness relationships) and the occurrence of transfer in their use of (de)-accentuation. Specifically, only marked L1 patterns were transferred from L1 to L2, suggesting that markedness is an important factor in L2 prosodic learning and transfer. Additionally, transfer from L1 is particularly persistent in prosody and can explain L2 learners’ difficulties adopting a language-appropriate pitch range [e.g., Curtis and D’Esposito (2002); Mennen (2004), and Scharff-Rethfeldt et al. (2008)]. Research suggests that non-target-like prosody in a L2 plays an important and independent role in the perception of foreign accentedness and native-listener judgments of comprehensibility (Magen, 1998; Jilka, 2000; Trofimovich and Baker, 2006). Mennen (2015) argued that the relative difficulty of L2 prosody is influenced by L1 (Willems, 1982; Jilka, 2000; Grabe, 2004; Mennen et al., 2010) and is, to some extent, predictable from universal markedness (Rasier and Hiligsmann, 2007; Zerbian, 2015) and from universal developmental paths in L2 prosodic acquisition in which some segmental learning must occur before learning intonational characteristics, such as stress, rhythm, tone, tempo pauses, loudness and voice quality (Nolan, 2006; Li and Post, 2014; Mennen and De Leeuw, 2014).

The influence of L1 on the acquisition of L2 prosody is supported by findings that stress distinctions are difficult for speakers of non-stress languages to process and—especially—retain in memory (Beckman, 1986; Dupoux et al., 1997; Peperkamp and Dupoux, 2002), as are tone distinctions (Shen, 1989). Listeners whose L1 is a non-stressed, tonal language, such as Chinese, are accustomed to syllabic non-stressed tonal information. Therefore, L1 Chinese listeners of L2 English often do not show sensitivity to English prosodic distinctions, neither online nor in memory retrieval after listening. For instance, Pennington and Ellis (2000) found that L1 Cantonese learners of L2 English had a poor memory for prosodically signaled information, including focused contrasts.

### Why L2 Differences?

To the extent that L2 learners do not show native-like prosodic processing (e.g., exhibiting a contrastive-uncertainty pattern rather than a contrast-representation effect), a second question is *why* these differences exist. This issue relates to more general questions about constraints on L2 learning. Here, we consider two potentially relevant constructs: proficiency and working memory.

One possibility is that non-native-like prosodic processing of L2 reflects a lack of knowledge of the L2. The mapping between prosodic cues and semantic and discourse information varies across languages. Thus, non-native listeners of any given language may initially have little knowledge of intonational form and meaning, especially since they are less frequently taught in formal L2 instruction, but may gain them with experience. Furthermore, PA processing in an atonal L2 may implicitly activate tonal language speakers’ representations of lexical tones, which may result in interference given that PA and lexical tone serve different linguistic functions. Under this account, as L2 knowledge and proficiency increases, interference from L1 lexical tone may decrease and L2 learners might be expected to become more native-like in their prosodic processing. Supporting this, Lee and Fraundorf (2017) found that low- and moderate-proficiency learners of L2 English showed no effects of contrastive PAs whatsoever, whereas high-proficiency learners did. Nevertheless, even high-proficiency learners did not process PAs the same way that native speakers did, suggesting that proficiency may not be the only relevant factor.

An alternative possibility is that L2 prosodic processing is constrained by more general cognitive resources, such as WM. There are at least two reasons to hypothesize a role for WM in L2 PA processing. First, learners of L2 English may have to rely more on declarative knowledge. Ullman (2001, 2004), Pinker (1999), and Pinker and Ullman (2002) have hypothesized that both declarative and procedural memory contributes to native speakers’ language processing. Declarative memory refers to verbalizable knowledge, for instance, in the domain of language, the association of vocabulary items with their respective meanings. In contrast, procedural memory is used to learn and control skills and habits that are not recognized explicitly. In the domain of language, native speakers process structural information (e.g., prosodic structure in the current study) based on procedural memory. But for L2 learners, the declarative-procedural (DP) model claims that their processing of linguistic knowledge might rely more on declarative memory. Thus, L2 learners may have access, and use declarative knowledge about pitch accents, which relies on working memory.

Second, greater WM capacity may help with the general processing demands of L2 comprehension. Difficulty with phonetic distinctions, lower vocabulary size, lesser accumulated lexical familiarity, and unfamiliarity with idiomatic expressions all combine to make non-native comprehension of spoken language less efficient than comprehension by native listeners (Akker and Cutler, 2003). As a result, it can be difficult enough to keep up with lexical and syntactic processing in an L2 language, leaving L2 comprehenders with insufficient processing resources for discourse or prosodic processing. Whereas native listeners can adopt a top-down mechanism in which their prosodic processing assists phonetic identification and lexical access, non-native listeners mainly employ a bottom-up mechanism in that they focus on the lexical and phonetic levels of information before applying prosodic cues (Akker and Cutler, 2003). Lee and Fraundorf (2017) suggested this could create the contrastive-uncertainty effect if L2 comprehenders do not have sufficient processing resources (i.e., WM) to encode which member of the contrast set is the true proposition and which is the contrastive alternative. Supporting this, individual differences in WM constrain PA interpretation even in L1 (Fraundorf et al., 2010).

Lastly, another possibility is that L1–L2 differences in prosodic processing are intrinsic to listening in a second language. This possibility accords with a long theoretical tradition proposing that L2 processing is qualitatively different from L1 processing even with extensive experience (Beckman, 1986; Cutler et al., 1997). In this account, neither greater working memory nor increased proficiency may be sufficient to modulate L2 PA processing.

### Research Questions and Hypotheses

In the present study, we tested how PAs influenced L1 Chinese learners’ memory for L2 English spoken discourse. We had two primary research questions:

First, we considered whether and how L2 learners’ ability to use PAs for encoding relevant contrasts in a discourse differs from that of native speakers. We contrasted the predictions of three hypotheses. The granularity hypothesis predicts that a contrastive PA should help rule out any false information. The contrast representation hypothesis predicts that a contrastive PA should specifically facilitate rejections of the false alternative. Finally, the contrastive uncertainty hypothesis predicts that a contrastive PA leads comprehenders to affirm both the correct item and (erroneously) the contrast item, but it does help them rule out the unmentioned item that is entirely outside the contrast set.

Second, we considered how prosodic processing varies with L2 proficiency and/or WM capacity. The theoretical rationale for exploring the cognitive factors in bilingual processing is crucial. We hope to reveal whether and to what extent the bilinguals with high proficiency or high cognitive control abilities (represented by working memory) could use PAs for encoding relevant contrasts in a discourse differs from that of native speakers. Although we already know that the native L1 speakers could clearly put information about PA in their text memory, we still do not know how text memory would be represented when there is uncertain information in the discourse. Therefore, we would predict again that a contrastive PA might leads bilinguals with more advanced cognitive abilities to affirm both the correct item to a certain extent but not in the confirmed L2 text memory and might cause them to erroneously remember the contrast item, and this effect might help them rule out the unmentioned item that is entirely outside the contrast set.

## Materials and Methods

### Participants

We recruited forty-two participants from each of four different subject populations of native Chinese speakers whom we expected to vary in English proficiency: high school students (Mean Age = 17.1, SD = 0.89), university undergraduates not majoring in English (Mean Age = 18.3, SD = 0.92), undergraduates majoring in English (Mean Age = 18.2, SD = 0.79), and graduate students of English (Mean Age = 21.4, SD = 0.67). The students were recruited from the similar educational backgrounds in the same neighborhood. Although there might be differences between the high school students and the university students in Chinese fluency, the differences were not that salient between these groups (*p* > 1). Their language ability differed only in English L2 fluency, rather than something more like maturation. All told, one hundred sixty-eight native speakers of Chinese (35 males) participated in the study. They were all recruited from the Beijing University of Science and Technology, from which the ethic committee approved the study in 2016 and the study was conducted in 2018 while the first author was sponsored by the Sino-US-Fulbright Scholarship. All resided in China at the time of participation.

To verify the differences in proficiency, all participants completed (a) a demographic survey on their language background reporting their years of formal English education and language proficiency and (b) the Quick Placement Test (Quick Placement Test [QPT], 2001), a test of English language proficiency (Geranpayeh, 2003). These two scores of years of formal English education and self-ratings of proficiency were highly correlated: η^2^ = 0.71, *p* < 0.05, suggesting that we had obtained reliable measures of participants’ English proficiency. Further, an ANOVA revealed that the proficiency level varied significantly across the four participant groups, *F*_(1,41)_ = 7.794, *p* = 0.003, η^2^ = 0.328, confirming that we had successfully identified groups that differed in their L2 proficiency.

Table 1 presents the demographic information as well as the average scores on the working memory tasks (discussed below) for each group. The reliability coefficients of the two WM measures were 0.69 and 0.72 respectively.

**TABLE 1:** Demographic information for the four participant groups.

Group	Age		Gender (M/F)	OSpan		RSpan		QPT		QPT proficiency percentile	Subject population	Years of English education
	*M*	SD		*M*	SD	*M*	SD	*M*	SD			
Low	16.73	1.2	23/19	13.01	3.6	10.34	3.9	28.45	3.25	<25th	High school students	8
Medium	19.58	2.2	4/38	11.44	3.7	9.08	3.3	37.65	3.84	25th- 50th	Non-English Major Undergraduates	10
Advanced	24.10	2.0	4/38	11.48	4.0	9.55	3.2	41.23	4.13	50th to 75th	English-major Undergraduates	11
High advanced	22.97	1.8	3/38	11.88	4.2	10.26	3.9	47.86	2.97	>75th	English postgraduates	14

*OSpan, operational span; RSpan, read span; QPT, quick placement test.*

### Materials

The listening materials were the 36 audio-recorded discourses previously used in Experiment 3 of Fraundorf et al. (2010) and Lee and Fraundorf (2017). Each discourse consisted of a context passage, such as (1a) below, introducing two contrastive sets of items (e.g., *British* and *French* as one set and *Malaysia* and *Indonesia* as the other), followed by a continuation passage that referred to one item in each contrast set, as in (1b). All these discourses were recorded by a native English speaker who was trained to produce the different pitch-accent types.

In a within-participants design, we varied whether the critical word in the continuation passage was produced with a presentational H* accent (indicated by regular text in the example) or a contrastive L + H* accent (indicated in capital letters in the example). The accent on each of the two critical nouns was orthogonally manipulated such that an L + H* accent could be placed on either the first critical word, second critical word, both, or neither. Audio recordings were created using cross-splicing such that only the critical words varied across conditions, and the rest of the recordings were identical.

(1a) Context Passage: Both the British and the French biologists had been searching Malaysia and Indonesia for endangered monkeys.
(1b) Continuation Passage: Finally, the (British/BRITISH) spotted one of the monkeys in (Malaysia/MALAYSIA) and planted a radio tag on it.

Memory for the spoken discourses was tested using probe statements presented *via* text, for which responses consisted of true/false. No prosodic cues were present during the test phase. Each critical word could be tested with one of three probe types: the correct fact, the contrastive alternative, or a wholly unmentioned item. For example, probes (2a) through (2c) test the critical word *British* in discourse (1), and probes (3a) through (3c) test the critical word *Malaysia*. Each critical word was tested in only one probe condition per subject.

(2a) The British scientists spotted the endangered monkey and tagged it.
(2b) The French scientists spotted the endangered monkey and tagged it.
(2c) The Portuguese scientists spotted the endangered monkey and tagged it.
(3a) The endangered monkey was finally spotted in Malaysia.
(3b) The endangered monkey was finally spotted in Indonesia.
(3c) The endangered monkey was finally spotted in the Philippines.

This resulted in a 4 × 2 × 3 factorial design: Proficiency Group (low, medium, advanced, and most advanced) × Pitch Accent Type (H* or L + H*) × Probe Type (correct, contrast, or unmentioned), with the first variable varying between subjects and the others within subjects. Assignment of items to conditions was counterbalanced across six presentation lists using a Latin Square design. The complete lists of stories and test probes are available in Fraundorf et al. (2010).

### Procedure

Participants completed the demographic questionnaire and QPT followed by two working-memory tasks before proceeding to the discourse-memory task.

#### Operation Span

To assess the participants’ working memory capacity (WMC), we implemented a version of the operation span (OSpan) task distributed by Redick and Engle (2006). The OSpan task has been shown to correlate with a wide range of higher-order cognitive tasks, such as reading and listening comprehension (Engle, 2010).

In the OSpan task, participants were presented with a series of simple equations one at a time. The left-hand side of each equation consisted of two operations, either (a) two additions or subtractions or (b) one addition/subtraction and one simple multiplication, such as 23–16 + 7 = 14? or 136 + 64 × 2 = 401? Participants had to mentally determine whether the number of the right-hand side of the equation correctly completed the equation and then responded by saying “yes” or “no” aloud. The experimenter pressed a key after the participant gave the response. Immediately afterward, a capital letter was displayed on the screen, which participants were tasked with remembering, and then the next equation appeared. The set of possible letters included only the *letters H*, *J*, *K*, *L*, *N*, *P*, *Q*, *R*, *S*, *T*, and *Y* because those letters were relatively phonologically distinct.

After a certain number of letters had been presented (the *span length*), participants were instructed to write down on paper the presented letters in the order in which they were presented. Two trials were presented at each of the span lengths from 2 to 9 sentence/letter pairs (14 trials total); the order of the trials was random. The task took approximately 15 min to complete.

Scoring was performed using *partial-credit unit scoring*, which in past work has been recommended for producing the most normal distribution of scores (Conway et al., 2005). If participants remembered all the letters in a particular trial, that was scored as 1. If participants remembered some but not all of the letters, they received a credit equal to the proportion of letters they did recall; for example, remembering two letters on a trial of span length six would be scored as 0.33. The maximum score for the task was 14.

#### Reading Span

We also implemented a modified version of the reading span (RSpan) task in Chinese (Daneman and Carpenter, 1980) modeled after the OSpan task. The reading span task was similar to the OSpan except that, instead of verifying equations, participants read aloud sentences in Chinese (e.g., [translated English version] *On warm sunny afternoons, I like to walk in the park.*?) and verified whether the sentence made sense by saying “yes” (makes sense) or “no” (does not make sense) immediately after they finished reading the sentence. As in the OSpan task, the to-be-remembered stimuli were capital letters presented between the sentences. We use a Chinese version of this task because we wanted this task to capture differences in working memory *per se* rather than L2 English proficiency, which we measured separately.

As in the OSpan task, the span length varied from 2 to 9 sentence/letter pairs, with two trials at each span length; the order of the same lengths was random. At the end of the series, participants wrote down the sequence of capitalized letters in the same order they read in the test. The RSpan task was scored the same way as the OSpan task, with a maximum score of 14.

#### Discourse Recognition Memory Task

Pilot testing indicated that remembering the discourses was relatively difficult for our L2 English participants. To reduce the memory burden and maximize the chance that performance was not at floor level, we split the materials into six blocks; each contained four recorded discourses followed by eight critical probe questions (two for each discourse) and two filler probes. The filler probes tested memory for other aspects of the stories unrelated to those emphasized by the PAs.

Each block began with the presentation of the auditory stimuli. During each story, the screen was black. There were a 5 s interstimulus interval between stories. After all of the stories in a block had been presented, the recognition memory task began. Each probe statement was displayed on the screen one at a time in different randomized orders from the original presentation, and participants indicated whether they judged the probe as *True* or *False* by pressing one of two keys on the keyboard. Participants were instructed to reject a probe as *False* if any part of it did not match the story they had heard. After participants made their response, there was a 1,000 ms interstimulus interval before the next probe was presented. Once all of the probes in a block had been presented, the procedure repeated with the study phase of the next block.

## Results

### Analytic Strategy

In the recognition memory task, the accurate answer to the correct probes was *true* but *false* to the other probe conditions. Thus, comparing simple accuracy rates across conditions confounds the accuracy of participants’ memory with any overall tendency to respond *true* or *false*. To eliminate this confound, we analyzed the memory data based on the theory of signal detection, in which the dependent measure is whether a participant judged a particular probe as *true* [Green and Swets, 1966; Macmillan and Creelman, 2005; for applications to mixed-effects modeling, Wright et al., 2009 and Murayama et al. (2014)]. This analysis allows a theoretical and empirical separation of *response bias* (an overall tendency to respond *true*) from *sensitivity* (the ability to discern whether a specific probe is *true*). Specifically, if participants have accurate memory for the discourse, they should respond *true* more often to the correct probes and less often to the contrast and unmentioned probes.

**FIGURE 1:**
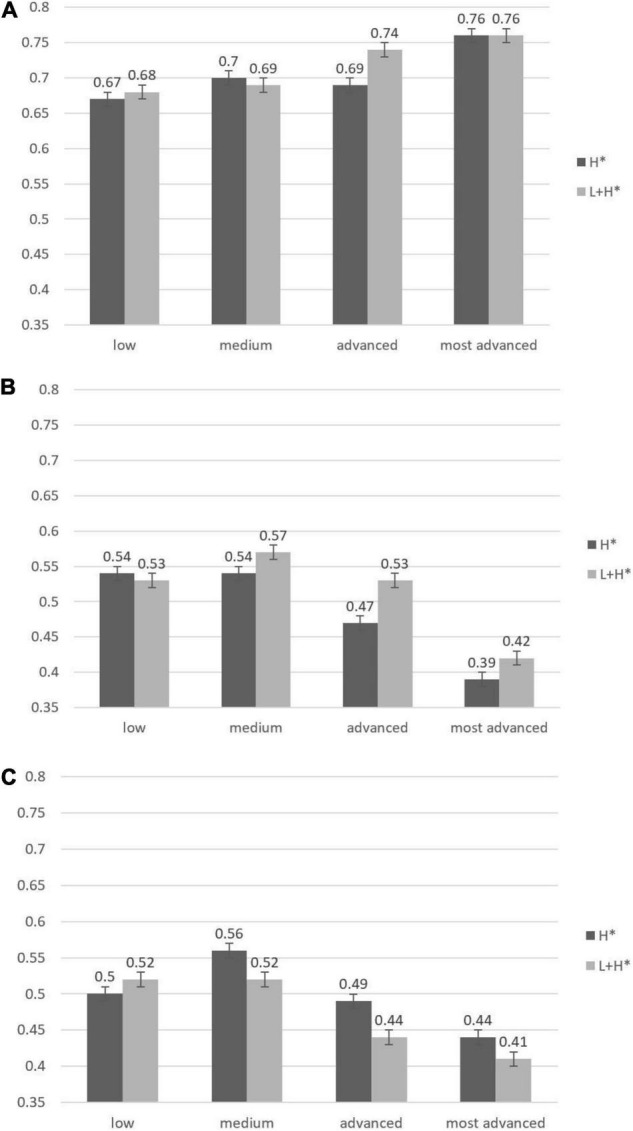
Bar charts of proportions of *true* responses across groups and conditions. (Note that *true* is a correct response to correct probes, but an incorrect response to contrast and unmentioned probes). **(A)** Correct probes. **(B)** Contrast-probes. **(C)** Unmentioned probes. *stands for the abbreviation.

We analyzed participants’ *true* responses in a mixed-effects logit model as a function of three fixed effects: pitch accent type, probe type, and proficiency level. All variables were coded with mean-centered contrasts to obtain estimates of main effects analogous to those from an ANOVA. For probe type, we used two effect-coded contrasts: One compared the responses to contrast probes to the mean rate of *true* responses, and one compared responses to unmentioned probes to the mean rate of *true* responses (Fraundorf et al., 2013) (Because our primary interest was in how participants rejected false information about the discourse, we were less interested in responses to correct probes, which essentially constituted fillers). The four ordered proficiency groups were coded using Helmert contrasts, which compares each successive proficiency group to the mean of the less proficient groups (e.g., advanced-proficiency learners versus medium- and low-proficiency learners). Figure 1 displays the mean rate of *true* responses for each proficiency group in each experimental condition, and Table 2 displays the results of the mixed-effects model.

**TABLE 2:** Fixed effects estimates from mixed logit model of “*True*” responses with probe type, accent type and proficiency group as fixed effects.

	Estimate	SE	Wald *z*	*p*-value
**Main effects across proficiency levels**				
Baseline rate of *true* responses (response bias)	0.29	0.06	5.59	< 0.001
Contrast probe vs. baseline (sensitivity)	–0.60	0.06	–10.81	< 0.001
Unmentioned probe vs. baseline (sensitivity)	–0.70	0.06	–12.62	< 0.001
L + H* accent (effect on response bias)	0.02	0.04	0.54	0.59
L + H* accent × contrast probe (effect on sensitivity)	0.17	0.11	1.50	0.13
L + H* accent × unmentioned probe (sensitivity)	–0.23	0.11	–2.05	0.04
**Effects of proficiency**				
Medium vs. low proficiency (response bias)	0.12	0.07	1.63	0.10
Advanced vs. low/medium proficiency (response bias)	–0.09	0.06	–1.47	0.14
Most advanced vs. low/medium/advanced proficiency (response bias)	–0.17	0.07	–2.46	0.01
Medium vs. low proficiency × contrast probe (sensitivity)	–0.01	0.15	–0.06	0.95
Medium vs. low proficiency × unmentioned (sensitivity)	–0.01	0.15	–0.04	0.96
Advanced vs. low/medium proficiency × contrast probe (sensitivity)	–0.19	0.13	–1.47	0.14
Advanced vs. low/medium proficiency × unmentioned (sensitivity)	–0.34	0.13	–2.64	0.01
Most advanced vs. low/medium/advanced proficiency × contrast probe (sensitivity)	–0.68	0.15	–4.66	< 0.001
Most advanced vs. low/medium/advanced proficiency × unmentioned (sensitivity)	–0.34	0.15	–2.24	0.02
**Effects of proficiency in comprehension of prosody**				
Medium vs. low proficiency × L + H* accent (response bias)	–0.04	0.10	–0.36	0.72
Advanced vs. low/medium proficiency × L + H* accent (response bias)	0.10	0.09	1.08	0.28
Most advanced vs. low/medium/advanced proficiency × L + H* accent (response bias)	–0.04	0.10	–0.43	0.69
Medium vs. low proficiency × L + H* × contrast probe (sensitivity)	0.42	0.29	1.45	0.15
Medium vs. low proficiency × L + H* × unmentioned probe (sensitivity)	–0.36	0.29	1.23	0.22
Advanced vs. low/medium proficiency × L + H* × contrast probe (sensitivity)	0.23	0.26	0.89	0.37
Advanced vs. low/medium proficiency × L + H* × unmentioned probe (sensitivity)	–0.52	0.26	–2.04	0.04
Most advanced vs. low/medium/high proficiency × L + H* × contrast (sensitivity)	0.12	0.29	0.40	0.69
Most advanced vs. low/medium/high proficiency × L + H* × unmentioned (sensitivity)	–0.01	0.29	–0.04	0.97

*stands for the abbrevation.

### Effects of L2 Proficiency

First, we examine overall trends across proficiency groups. The positive intercept term indicates that, overall, participants had a bias to respond *true* rather than *false*, with the odds 1.33 (95% CI: [1.19, 1.50]) in favor of responding *true*. This bias to respond *true* was obtained despite the fact that the majority of probes were false; that is, participants often accepted false statements. Nevertheless, participants responded *true* less frequently to contrast probes and unmentioned probes, indicating that they had at least some veridical memory for the discourse. Specifically, the odds of responding *true* were reduced 1.82 times (95% CI: [1.62, 2.05]) for contrast probes and 2.01 times (95% CI: [1.79, 2.27]) for unmentioned probes.

### Effects of Pitch Accents

What about the effects of PAs? Pitch accent type had no main effect on response bias, indicating that contrastive PAs did not simply induce an overall bias to respond *true* or *false*. Rather, pitch accent interacted with probe type: For probes referring to items unmentioned in the discourse, the odds of correct rejection increased by 1.26 times (95% CI: [1.01, 1.56]) when the critical word was originally heard with a contrastive L + H* pitch accent. By comparison, L + H* did not significantly facilitate rejection of the salient contrastive alternatives, consistent with the results of Lee and Fraundorf (2017); indeed, the effect was numerically (but non-significantly) in the direction of the L + H* accent *hindering* correct rejection.

### Pitch Accent Effects Qualified by Proficiency

Importantly, however, many of these effects varied across proficiency levels. First, the overall bias to respond *true* was 1.19 times smaller (95% CI: [1.03, 1.36]) for the most advanced learners; that is, at the most advanced proficiency level, participants had less of a tendency to simply accept the presented statements as true. Second, participants with more advanced proficiency were more accurate at judging whether specific probe statements were true. The odds of correctly rejecting an unmentioned probe were 1.40 times greater (95% CI: [1.09, 1.81]) for advanced-proficiency learners than low- or medium-proficiency learners, though advanced-proficiency learners were still no more successful at ruling out the salient contrast items. It was not until the high advanced level of proficiency that learners finally showed greater success in rejecting the contrast probes, with the odds of correct rejection increasing by 1.97 times (95% CI: [1.47, 2.65]) for the most advanced learners as compared to the other groups. The most advanced learners also showed a further 1.40-times increase (95% CI: [1.05, 1.89]) over the advanced learners in the odds of correctly rejecting the unmentioned probes.

Most critically, the effects of prosody were also qualified by proficiency. Specifically, for learners who had attained at least advanced proficiency, the benefit of the contrastive L + H* accent in rejecting the unmentioned probes was 1.68 times greater than for less proficient learners (95% CI: [1.01, 2.80]); high advanced learners did not further differ in this effect. As noted above, the L + H* accent did not affect participants’ overall tendency to respond *true* or *false*, and this did not interact with proficiency level; that is, at no proficiency level did the PAs affect response bias.

### Effects of Working Memory

We also examined whether apparent effects of proficiency reflect proficiency with the language itself or rather the ability to hold more material in working memory. Because we had two working-memory measures (which showed modest agreement, *r* = 0.46, *p* < 0.001), we created a composite measure by averaging each participant’s *z*-scores on each of the two tasks; using multiple measures in this way reduces measurement error by reducing the influence of task-specific variance associated with any particular task [e.g., the influence of arithmetic ability on OSpan performance; Cronbach (1957) and Bollen (1989)].

It seems unlikely that our present effects of proficiency can be attributed to working memory: More proficient learners did not necessarily have higher working memory; indeed, the Spearman correlation between proficiency rank and working memory was actually *negative*, rho = −0.31, *p* < 0.001, such that more proficient learners had *lower* working memory. Indeed, as can be seen in Table 1, the group that scored highest in working memory was the *low* proficiency group. Thus, it does not seem to be the case that the more proficient groups were more sensitive to contrastive prosody because they had greater working-memory resources.

Nevertheless, as a more direct test of whether working memory accounts for the proficiency effects, we replaced the proficiency variable in the mixed-effects model with the mean-centered working memory score^2^ to test whether working memory could be observed to have similar effects as proficiency.

Table 3 displays the results of the mixed-effects modeling including working memory. There was some evidence that participants with higher working memory scores performed better on the task overall. A 1-standard deviation increase in working memory corresponded to a significant 1.15 times (95% CI: [1.02, 1.29]) increase in the odds of successfully rejecting probes referring to items unmentioned in the discourse, and a marginal 1.13 times (95% CI: [1.00, 1.27]) in the odds of successfully rejecting the contrastive alternatives. Critically, however, working memory and the unmentioned text did not significantly interact with pitch accent type; there was no evidence that the benefit of L + H* accents in rejecting either contrast probes or unmentioned probes was enhanced for people with greater working memory (both *ps* > 0.90).

**TABLE 3:** Fixed effects estimates from mixed logit model of “True” responses with probe type, accent type, and working memory as fixed effects.

	Estimate	SE	Wald *z*	*p*-value
**Main effects across proficiency levels**				
Baseline rate of *true* responses (response bias)	0.30	0.05	5.66	< 0.001
Contrast probe vs. baseline (sensitivity)	–0.55	0.06	–10.01	< 0.001
Unmentioned probe vs. baseline (sensitivity)	–0.67	0.06	–12.10	< 0.001
L + H* accent (effect on response bias)	0.03	0.04	0.79	0.43
L + H* accent × contrast probe (effect on sensitivity)	0.15	0.11	1.38	0.17
L + H* accent × unmentioned probe (sensitivity)	–0.20	0.11	–1.85	0.06
**Effects of working memory**				
Working memory (response bias)	–0.03	0.03	–0.76	0.45
Working memory × contrast probe (sensitivity)	–0.12	0.06	–1.85	0.06
Working memory × unmentioned (sensitivity)	–0.14	0.06	–2.18	0.03*
**Effects of WM in comprehension of prosody**				
Working memory × L + H* accent (response bias)	0.03	0.05	0.58	0.56
WM × L + H* × contrast probe (sensitivity)	–0.01	0.13	–0.12	0.91
WM × L + H* × unmentioned probe (sensitivity)	> −0.01	0.13	> −0.01	0.99

*Significant level = 0.05.

## Discussion

The present study examined how L1 Chinese learners of L2 English processed and remembered spoken L2 discourses containing contrastive PAs (L + H*) or non-contrastive presentational PAs (H*). We tested L2 learners’ memory using a recognition task including three types of probes: correct, contrastive alternative, and unmentioned items. We also assessed the readers’ language proficiency (using two standardized English proficiency scores) and working memory (RSpan and OSpan).

We contrasted three hypotheses about how contrastive PAs might influence memory for a discourse: the granularity account, the contrast representation account, and the contrast uncertainty account. The granularity account predicts that the salient acoustic or perceptual aspects of a contrastive PA facilitate memory representations of the accented word itself, which should help comprehenders reject any items inconsistent with the true statement (Sanford et al., 2006). Alternatively, the contrast-representation hypothesis proposes that contrastive PAs, relative to presentational PAs, promote representation of a specific salient alternative and should facilitate rejection of only that salient alternative, not a completely unmentioned item; this pattern has been found for L1 English comprehenders (Fraundorf et al., 2010). Lastly, the contrastive uncertainty hypothesis (Lee and Fraundorf, 2017) proposes that because contrastive PAs evoke the salient alternative, they lead to confusion over which was the correct proposition and which was the salient alternative; this should allow comprehenders to easily reject the unmentioned items, but to have difficulty discriminating the correct and contrast items.

Our principal findings are threefold. First, across all proficiency levels, our L2 English learners did not show a native-like contrast-representation effect in which contrastive PAs facilitated rejections of a specific salient alternative in memory. Instead, to the extent PAs influenced L2 comprehenders’ memory at all, they showed a contrastive uncertainty pattern. Contrastive PAs helped L2 learners reject the unmentioned item that was never part of the discourse, but they impaired L2 learners’ ability to discriminate between the correct item and its salient alternative. This finding replicates previous studies among a different population of L2 English learners whose L1 was Korean (Lee and Fraundorf, 2017, 2021). Second, we found a significant interaction effect of proficiency by contrastive PA. Specifically, the benefit of contrastive PA in rejecting unmentioned items was enhanced for both advanced and high-advanced learners relative to low- or medium-proficiency learners. More proficient participants also showed more accurate memory for the discourses overall, as well as a reduced overall bias to affirm the presented statements as *true*. Third, there was no evidence that the benefit of contrastive PAs in rejecting either contrast probes or unmentioned probes was enhanced for people with greater working memory. We discuss the implications of each of these findings below.

### A Contrastive Uncertainty Effect in L2 Pitch Accent Comprehension

The current study indicates that contrastive PAs led L2 listeners to represent salient alternatives differently from L1 English native speakers. For native speakers, emphasizing a word with a contrastive PA helped listeners rule out a specific alternative to that word on a later memory test, suggesting that they had represented that particular alternative in memory. L2 learners did not derive these same memory benefits. Among the low- and mid-proficiency groups, there were no mnemonic benefits of contrastive accents whatsoever. Among more proficient learners, there was a different effect such that contrastive PAs facilitated rejection of items entirely unmentioned in the discourse. This pattern suggests that L2 learners may have represented the *set* of alternatives—which would help reject any item in the set—but failed to distinguish which was the true proposition and which was the salient alternative. Interestingly, the most advanced group revealed a somewhat better ability to rule out the contrastive alternative, but this was not qualified by PA type, suggesting that lexical tone may interfere with representations of English PA even in this group.

These results replicate those of previous studies of L1-Korean L2-English undergraduates (Lee and Fraundorf, 2017, 2021). Notably, however, we replicate them in a population with a different L1 (Chinese) and with a wider age range (from high school to graduate study). This suggests that the prior results were not simply an idiosyncratic effect in L1 Korean learners. Rather, a more general property of second-language processing may be difficulty in distinguishing the members of a set of alternatives.

This difficulty in distinguishing members of a contrast set may have been enhanced by the isolated nature of our stimuli. Theories of memory generally distinguish *episodic memory* for things and events that happened to a person from *semantic memory*, or more general knowledge (James, 1890). Although our materials were semantically coherent and comprehensible, these short, discrete stories were largely distinct from listeners’ prior semantic knowledge and primarily tapped episodic memory. This may have made it particularly difficult to distinguish the two members of the contrastive set (such as whether the *British* or *French* scientists found the monkey) because this relied entirely on detailed episodic information. But an irrelevant or unmentioned item, such as *Portuguese* scientists, could be more easily rejected since this piece of semantic memory did not exist in the memory trace.

### Proficiency-Driven Pitch Accent Effects on Memory Representation

We found modulation and qualification of PA effect by proficiency. Similar to previous research (Lee and Fraundorf, 2017, 2021), less proficient learners did not show any sensitivity to contrastive PAs whereas more proficient learners did—though even more proficient learners did not show fully native-like comprehension.

Why is proficiency critical to capitalizing on prosodic information in remembering a discourse? We speculate there at least two reasons. First, L2 proficiency can shape language processing and cognition more generally such that proficient L2 learners have better attentional focus, which supports veridical memory encoding and recognition. This attention advantage for bilinguals has been demonstrated consistently in the reading and second language learning literature (Bialystok, 2017). Thus, even though the L2 listeners in our study did not reach the highest level of native-L1-like performance, they have benefited over less proficient L2 learners in focusing attention and storing contrastive information more stably and steadily in memory.

Second, L2 proficiency may be critical to understanding the meaning of PAs themselves and to understanding how interference from L1 lexical tone affects their representation. Although we are unaware of any research examining the influence of a tonal L1 on L2 PA interpretation, the finding that contrastive PA failed to facilitate the most advanced L2 English learners’ ability to rule out contrastive alternatives suggests that L1 lexical tone may have interfered with their representations of L2 PA, preventing them from using it to strengthen memory for contrastive information in discourse as native speakers do. Native-like processing of English PAs requires L2 English learners to learn how particular PA types should be mapped to discourse representation. This linguistic knowledge may be acquired only gradually with increasing proficiency, especially since it is rarely taught in formal instructions. Consistent with this claim, Lee and Fraundorf (2019) suggest that L2 learners *can* make use of other cues to focus whose purpose may be more readily apparent, such as font emphasis.

### Working Memory Effects

We also observed a significant effect of WM in that WM predicted participants’ ability to correctly rule out the probe statements referring to items wholly unmentioned in the original discourse. This effect may be thought of in terms of familiarity (Yonelinas, 2002) or episodic memory traces. In example (1), since both *British* and *French* appeared in the discourse in some capacity, it may have been difficult to distinguish them. By comparison, the unmentioned lure *Portuguese* did not appear in the discourse at all and would have not existed in the memory trace as this part of the discourse wasn’t mentioned, so participants could have more confidently rejected it. Thus, performance on the task may be related to participants’ ability to retrieve details of the memory traces. This is in line with the argument that deficits in episodic memory are associated with reduced retrieval of episodic details and reduced coherence of discourse (Seixas-Lima et al., 2020). Another reason may be that the trace retrieval strategy of episodic memory promotes the long-term retention of bilingual vocabulary in the mind. This dovetails with the finding of Zhang et al. (2021) that the non-verbal episodic memory ability of highly proficient bilinguals contributes to bilingual vocabulary development. Nonverbal episodic memory skills contribute to lexical competence because participants with them become more proficient at higher levels.

Working memory may be especially important for L2 discourse comprehension (although WM also predicts baseline performance in discourse memory even for L1 participants; Fraundorf et al., 2012). As claimed by Witzel and Forster (2012), L2 words must be stored in working memory; however, L1 words are placed in the semantic systems that store knowledge. The link between episodic memory ability and L2 lexical competence suggests that episodic memory may play a role not only in initial second language lexical acquisition but also possibly in long-term retention and representation of second language vocabulary. These results are also consistent with the episodic L2 hypothesis (e.g., Jiang and Forster, 2001; Witzel and Forster, 2012), which predicts that episodic L2 lexical representations persist even at higher levels of bilinguals’ proficiency at later stages. Memory plays an important role in bilinguals’ L2 lexical repertoires. Furthermore, these results suggest that individual differences in working memory affect memory for L2 spoken discourse. L2 episodic memory is activated first, due to repetition of words (Witzel and Forster, 2012); second, due to prosodic feature adjustments, e.g., similarity of the talker in voice (Shao et al., 2017), segmental and suprasegmental features (Lengeris, 2012), or conjunction illusions (e.g., list 1 and list 2) (Brainerd et al., 2014).

Critically, however, while working memory predicted overall performance, we did *not* find that working memory moderated or qualified the use of the information conveyed by contrastive pitch accents: The pitch accenting effect was no larger (or smaller) for participants higher in working memory. This suggests that limits in the ability to carry out online cognitive operations were not the reason that L2 learners struggled to make use of the contrastive pitch accents (see also Lee and Fraundorf, 2021 for a similar conclusion).

### Significance and Practical Implications

What practical applications does this current study have? We found that L1 Chinese learners of L2 English did have some ability to leverage contrastive PAs in language comprehension, at least when they were relatively advanced in proficiency. This suggests that contrastive PAs can be useful in conveying information even to L2 listeners. Nevertheless, our results suggest that L2 learners may have limited and insufficient knowledge of the meaning of L2 intonation (as we discuss above). Thus, it may be beneficial to teach L2 learners how to attend to and interpret salient intonational information.

Second, methodologically, we capitalized on the use of mixed-effects models to address the problems of a non-normal dependent variable. These models permit the use of link functions, such as the log odds (known as the logit), to relate experimental or observational variables to outcome variables that are not normally distributed, such as binomial outcomes like recognition accuracy (Baayen et al., 2008; Jaeger, 2008). Further, by incorporating information from multiple levels—both trial-level characteristics of the experimental design and subject-level individual differences, such as working memory—and their interaction, we could examine how different types of English L2 learners leverage PA cues.

### Limitations and Future Directions

Because the discourses were presented aurally, one question is whether contrastive PA interpretation varied with proficiency effects simply because only more proficient learners could comprehend the lexical and syntactic content of the auditory input. This explanation may apply to some extent: Overall memory accuracy, regardless of PA type, increased with proficiency. Nevertheless, above and beyond these effects, we found effects of proficiency on how contrastive PAs affect memory.

Although our work suggests that comprehenders gradually learn how to map L2 PAs onto particular meanings with increasing proficiency, one question for future work is how, precisely, this mapping is acquired. The contrastive L + H* PA is acoustically more salient than the presentational H* PA, but earlier research among L1 native speakers suggested that the mnemonic benefit of contrastive PA stem from their contrastive interpretation and not merely its audibility or perceptual salience (Cutler et al., 1997; Fraundorf et al., 2010; Wagner and Watson, 2010). Therefore, future work could examine how perceptual features (such as embodied perceptual symbols) are integrated into the ultimate memory representation of the text (Barsalou, 1999; Del Giudice et al., 2004).

The current study is based upon L2 English proficiency, and we did not assess the students’ L1 Chinese language proficiency and fluency. Thus, it’s possible that a confound between Chinese and English ability may serve as an alternative explanation if the pitch accents that participants heard were easy to relate to their Chinese L2 native language.

Finally, although recent research suggests that L2 learners tend to be more sensitive to online sentence processing as their WM capacity in L1 increases (Coughlin and Tremblay, 2013), we did not observe any such effects. One reason for this may be that WM was inversely related to proficiency within our sample—WM scores were highest among the low-proficiency group—which might have obscured any potential WM effects. Future research could more thoroughly investigate this issue by examining variability in WM within L2 speakers with similar proficiency.

## Conclusion

We examined that how PAs influenced how L1-Chinese learners of L2 English comprehended and remembered a spoken L2 discourse. We compared four L2 proficiency groups (low, medium, advanced, and high advanced) based on their Quick Placement Text (QPT) levels. Signal detection analysis (implemented via mixed-effects modeling) revealed that L2-English learners were more sensitive to PA as L2 proficiency increased. However, even the most advanced learners showed a pattern of memory effects distinct from native speakers: Rather than discriminating a correct proposition from a salient alternative in the discourse, contrastive PAs facilitated rejection only of items never mentioned in a discourse, suggesting that L2 learners had difficulty discriminating the items within contrast sets. Further, these effects were influenced only by proficiency and not by WM, suggesting they reflect incomplete knowledge of the intonation-to-meaning mapping more than limitations in online processing resources.

## Data Availability Statement

The raw data supporting the conclusions of this article will be made available by the authors, without undue reservation.

## Ethics Statement

The studies involving human participants were reviewed and approved by University of Science and Technology. The patients/participants provided their written informed consent to participate in this study.

## Author Contributions

CG and SF designed the study. CG conducted the research. SF analyzed the data. All authors wrote the manuscript and approved the submitted version.

## Conflict of Interest

The authors declare that the research was conducted in the absence of any commercial or financial relationships that could be construed as a potential conflict of interest.

## Publisher’s Note

All claims expressed in this article are solely those of the authors and do not necessarily represent those of their affiliated organizations, or those of the publisher, the editors and the reviewers. Any product that may be evaluated in this article, or claim that may be made by its manufacturer, is not guaranteed or endorsed by the publisher.

## Footnotes

1 Lee and Fraundorf (2017) called this a shallow representation; here, we introduce the term *contrastive uncertainty* to better distinguish this account of the effect of PAs from the (largely unrelated) Shallow Structure Hypothesis account of L2 syntactic processing.

2 We use each participant’s individual working memory score rather than splitting participants into discrete groups because this preserves the full range of variation in working memory and contributes more information and more statistical power to the model (Cohen, 1983).
